# Psychische Belastung während der COVID-19-Pandemie: Konsequenzen für psychiatrisch Erkrankte und therapeutische Implikationen

**DOI:** 10.1007/s00115-020-01056-y

**Published:** 2021-01-12

**Authors:** Johanna G. Winkler, Dario Jalilzadeh Masah, James Kenneth Moran, Joachim Bretz, Ioannis Tsagkas, Thomas Goldschmidt, Meryam Schouler-Ocak

**Affiliations:** 1 Psychiatrische Institutsambulanz der Charité im St. Hedwig-Krankenhaus, Charité – Universitätsmedizin Berlin, Große Hamburger Str. 5–11, 10115 Berlin, Deutschland; 2 Research Group Multisensory Integration der Charité im St. Hedwig-Krankenhaus, Charité – Universitätsmedizin Berlin, Berlin, Deutschland

**Keywords:** COVID-19-Pandemie, Psychische Erkrankung, Psychische Belastung, Lockdown, Krisenintervention, COVID-19 pandemic, Mental disorder, Psychological stress, Lock down, Crisis intervention

## Abstract

**Hintergrund:**

Der Ausbruch der COVID-19-Erkrankung und die rasche Ausbreitung des sie verursachenden Coronavirus SARS-CoV‑2 bedroht weltweit nicht nur die physische, sondern auch die psychische Gesundheit der Bevölkerung. Seine Auswirkungen auf Neuerkrankungen und die Entwicklung bestehender Erkrankungen im ambulanten psychiatrischen Bereich in Deutschland ist noch nicht untersucht.

**Methoden:**

Die Dokumentation in den Akten von 682 behandelten psychisch erkrankten Personen wurde retrospektiv bezüglich ihrer subjektiv erlebten Belastung durch die Pandemie und der klinischen Relevanz hin untersucht.

**Ergebnisse:**

Bei 60,5 % (*n* = 378) bestand eine psychische Belastung durch diese Pandemie. 14,5 % (*n* = 99) der Betroffenen litten unter Angst vor dem Virus SARS-CoV‑2 und einer möglichen Infektion, 25,5 % (*n* = 174) unter den getroffenen Schutzmaßnahmen (Lockdown) und 4,3 % (*n* = 29) unter beidem; hierbei bestanden signifikante diagnoseabhängige Unterschiede. Angsterkrankte waren signifikant stärker belastet und hatten mehr Angst vor der Erkrankung, Psychoseerkrankte waren signifikant weniger belastet. Bei 43,7 % aller dieser Belasteten (*n* = 132) wurde eine akute therapeutische Intervention erforderlich, 6,0 % (*n* = 18) mussten stationär eingewiesen werden.

**Diskussion:**

Psychisch Vorerkrankte gehören zu den durch die Belastungen der COVID-19-Pandemie besonders gefährdeten Bevölkerungsgruppen. Langfristigere Untersuchungen zum Verlauf der psychischen Belastungen unter Pandemiemaßnahmen sowie Studien zur Förderung von Resilienz in dieser Bevölkerungsgruppe und die Implementierung solcher Maßnahmen sind erforderlich.

## Abstract (en)

**Background:**

The outbreak of the COVID-19 disease and the rapid spread of the inducing coronavirus SARS-CoV‑2 threatens not only people’s physical health but also their mental health. Its influence on incidence and course of existing illnesses in the psychiatric outpatient sector in Germany is still unknown.

**Methods:**

The medical reports of 682 persons in psychiatric treatment were retrospectively investigated, regarding their subjective response to this pandemic and its clinical relevance.

**Results:**

Of the patients 60.5% (*n* = 378) experienced greater psychological stress, 14.5% (*n* = 99) reported fear of the SARS-CoV‑2 and the possible danger of infection, 25.5% (*n* = 174) reported fear resulting from the protective measures taken (lock down) and 4.3% (*n* = 29) reported fear of both. This differed significantly across diagnoses: people with anxiety disorders reported significantly greater stress as well as greater fear of the virus, whereas people with psychoses were significantly less affected than the other patients. Of the participants 43.7% (*n* = 132) were so strongly affected that acute treatment had to be implemented and 6.0% (*n* = 18) had to be referred to inpatient care.

**Discussion:**

People with mental illnesses are particularly vulnerable to the psychological strain of the COVID-19 pandemic. Long-term studies on the further course of disease will be necessary. Additional studies that test interventions to build resilience in this population will also be needed.

Die globale Ausbreitung und individuelle Bedrohung durch die COVID-19-Pandemie und die zur Bewältigung dieser Krise getroffenen gesetzlichen Maßnahmen stellen jede*n Einzelne*n vor individuelle psychische Herausforderungen. Sowohl die Angst vor einer Ansteckung mit dem Virus oder der Erkrankung geliebter Menschen als auch die Folgen der Hygienemaßnahmen mit Kontaktbeschränkungen bis hin zur Isolation sind sehr belastend und lassen allgemein die Entwicklung psychischer Erkrankungen befürchten. In diesem Beitrag werden die psychischen Konsequenzen für psychisch erkrankte Personen untersucht.

Psychische Folgen individueller lebensbedrohlicher Situationen sind vorbeschrieben [37, 43]. Ebenso sind psychische Erkrankungen als Folge kollektiver und globaler Bedrohungen bekannt – z. B. bei Naturkatastrophen [33], im Krieg [16] oder auf der Flucht [18]. In dieser aktuellen Pandemie werden weltweit vermehrt psychische Erkrankungen beobachtet. In China wurden bei 12- bis 18-jährigen Jugendlichen hohe Raten an krankheitswertiger ängstlicher (43,7 %) und depressiver (37,4 %) Symptomatik gemessen, wobei beides negativ assoziiert war mit fachlichem Wissen über das Virus [42]. Auch krankheitswertige Symptome einer posttraumatischen Belastungsstörung (PTSD) wurden beobachtet – in Italien [7] und China [40] bei rund einem Drittel der Allgemeinbevölkerung, in den USA bei 31,8 % [20]. Frauen sind besonders belastet [7, 8].

In der COVID-19-Pandemie hat soziale Isolation einen schützenden Effekt bezüglich Ansteckungswahrscheinlichkeit und Mortalität und somit für die physische Gesundheit [26]. In der Allgemeinbevölkerung bewirkt soziale Isolierung im psychischen Bereich eine Zunahme von depressiven, Angst- und Stresssymptomen sowie Ärger und wirkt u. U. stigmatisierend [28].

Für einzelne Personengruppen wurde die psychische Vulnerabilität in dieser Situation bereits gesondert betrachtet. In Kanada wird die besondere klinische Beobachtung schwangerer Frauen angedacht, die aktuell wesentlich mehr mit ängstlich-depressiven, dissoziativen und posttraumatischen Symptomen belastet sind als vor der Pandemie [3]. Weltweit wurde vermehrt psychisch und physisch traumatisierende häusliche Gewalt als unmittelbare Folgen eines Lockdowns beobachtet [13, 22]; infolgedessen werden bereits spezielle Schutzmaßnahmen für Frauen [27] und Kinder [21] gefordert. Auch Gesundheitsbeschäftigte wurden spezifisch untersucht [25], insbesondere das auf Intensivstationen tätige Personal, das schon in Vor-Corona-Zeiten psychisch sehr stark beansprucht war [14].

Eine weitere, bislang wenig beachtete, für psychische Belastung sehr gefährdete Gruppe der Bevölkerung ist die der psychisch Vorerkrankten. Bei ihnen besteht eine signifikant stärkere und häufigere Entwicklung von u. a. Suizidgedanken, PTSD-Symptomatik und Schlafstörungen als bei der psychisch gesunden Allgemeinbevölkerung [15]. Unter diesen sind die älteren Menschen mit somatischen Komorbiditäten und die sozioökonomisch Gefährdeten besonders betroffen [6]. Eine Zunahme von Alkoholrückfällen und Wahnsymptomatik wurde vermutet [17, 31, 35].

Die psychiatrische Akutversorgung müsse sich auf die Behandlung schwer psychisch erkrankter SARS-CoV-2-Infizierter vorbereiten [12], als Reaktion auf die täglich wechselnden Hygienemaßnahmen bestünde die Notwendigkeit, „fast täglich die Psychiatrie neu“ zu „erfinden“ [31], ähnlich wie in der Psychiatrischen Universitätsklinik der Charité im St. Hedwig-Krankenhaus umgesetzt (PUK der Charité im SHK; [17]). Faktisch bedeutete die COVID-19-Pandemie-bedingte Umstrukturierung der psychiatrischen Akutstationen, dass mehr schwer psychisch Erkrankte auf weniger stationäre Behandlungsplätze trafen und daher ambulant mitbehandelt werden mussten. Dies in einer Zeit, in der komplementäre ambulante Betreuungsangebote (z. B. Tageskliniken, Tagesstätten, Werkstätten, Einzelfallhilfe, Soziotherapie, ergotherapeutische Gruppen) zum großen Teil nicht zur Verfügung standen [10].

Wir nahmen an, dass die von uns behandelten psychisch erkrankten Menschen im Rahmen der COVID-19-Pandemie erheblich psychisch belastet sein würden, wie dies andere Studien sowohl für die Allgemeinbevölkerung [7, 20, 40, 42] sowie auch für die psychisch vorerkrankte Bevölkerungsgruppe beschrieben [6, 15]. Wir erwarteten diagnoseabhängig unterschiedliche Aussagen bezüglich einer Belastung durch Infektionsangst oder die Folgen der Schutzmaßnahmen sowie eine diagnoseabhängige Prävalenz beobachteter klinischer Symptomatik. Dabei erwarteten wir zudem vermehrte Trinkrückfälle bei Alkoholabhängigen und Wahnsymptomatik bei schizophren Erkrankten [35], aber auch – wie in anderen Studien vorbeschrieben – eine stärkere Zunahme von Angst, Depression, Ärger, Impulsivität, Schlafstörungen, Suizidgedanken [9] und PTSD-Symptomatik [7, 15, 20, 40]. Ebenso vermuteten wir eine höhere Belastung bei älteren Behandelten [6] und Frauen [7, 8].

Wir bildeten die Hypothese, dass es sowohl für die klinische Verschlechterung der Symptomatik als auch für die subjektive Wahrnehmung der belastenden Ursache diagnoseabhängige Unterschiede geben wird.

## Methodik

In einer retrospektiven Analyse untersuchten wir die Dokumentationen in Krankenakten aller von uns während der Berliner Lockdown-Maßnahmen im Zeitraum vom 01.04.2020 bis zum 30.04.2020 ambulant Behandelten hinsichtlich Geschlecht und Alter, psychischer Erkrankungen, der klinischen Veränderung ihrer Krankheitssymptomatik, der subjektiv wahrgenommenen Ursache dieser Veränderung und der von uns getroffenen therapeutischen Maßnahmen. Dabei berücksichtigen wir die von uns in einem Pflegeheim Behandelten nicht, da sehr viele von diesen krankheitsbedingt nicht mehr zur differenzierten verbalen Kommunikation fähig waren.

Bei den Daten der vorliegenden Studie handelt es sich um eine Sekundärdatenanalyse [2, 32, 41] der vorliegenden Krankenhausdaten der Psychiatrischen Institutsambulanz der Charité im SHK Berlin. Die Krankheitssymptomatik der behandelten Patient*innen wird nicht nur einmalig, sondern bei jedem ihrer Besuche in der psychiatrischen Institutsambulanz dokumentiert.

Damit liegen nicht alleine Daten während der Berliner Lockdown-Maßnahmen im eigentlichen Beobachtungszeitraum vom 01.04.2020 bis zum 30.04.2020 vor, sondern auch Daten für den Zeitraum vor Beginn der Lockdown-Maßnahmen. Daher kann die Veränderung der Symptomatik erfasst werden, indem die Daten vor den Lockdown-Maßnahmen mit den Daten während der Lockdown-Maßnahmen verglichen werden. Die vorliegenden Daten (vor Lockdown, während Lockdown) wurde von unserer Arbeitsgruppe geratet [4]. Unterschieden wurden drei Kategorien, in denen sich die Intensität der klinischen Verschlechterung bei einem Patienten abbildet. Unterschieden wurde, ob sich „keine“, eine „leichte“ bzw. eine „schwere“ Verschlechterung zeigt. Standardisierte Fragebögen wurden nicht verwendet, da sich die dafür erforderlichen detaillierteren Aspekte nicht extrahieren ließen.

Dabei wurden die in der von den Behandelnden in der Krankenakte dokumentierten pathologischen Verhaltensänderungen – wie z. B. starker sozialer Rückzug, Suizidimpulse oder -handlungen, stärkeres Konsumverhalten – als „schwere klinische Verschlechterung“ gewertet, während die ausschließlich im pathologischen Befund beschriebenen Verschlechterungen – wie z. B. Zunahme des Grübelns, Auftreten von Suizidgedanken usw. – als „leichte klinische Verschlechterung“ gewertet wurden. Standardisierte Fragebögen wurden nicht verwendet. Die Äußerungen der Patient*innen bezüglich der subjektiv wahrgenommenen Belastung wurden in den Kategorien „nicht belastet“, „Belastung durch die Angst im Zusammenhang mit SARS-CoV‑2 und COVID-19 (‚durch Angst vor Virus‘)“, „Belastung durch eine der direkten oder indirekten Konsequenzen der gesetzlich implementierten Hygienemaßnahmen (‚durch Maßnahmen‘)“, „es geht mir besser als sonst (‚es geht mir besser‘)“, „Belastung sowohl durch Angst vor Infektion als auch durch Maßnahmen (‚durch beides‘)“, „andere, nicht durch die Pandemie bedingte Belastung (‚durch etwas anderes‘)“ oder „fehlende Angabe“ unterschieden.

Die therapeutischen Maßnahmen wurden von den psychiatrisch erfahrenen Behandelnden, ggf. unter Supervision, entschieden, die Diagnosen nach ICD-10 gestellt. Da sich die Haupt- und Nebendiagnosen systembedingt nicht unterscheiden ließen, berücksichtigten wir pro Patient*in bis zu zwei Diagnosen unterschiedlicher Syndromzugehörigkeit (häufbare Merkmale) in unserer Bewertung, sodass ein depressiver, alkoholabhängiger Patient sowohl im Syndromcluster „Abhängigkeit“ als auch im Cluster „depressives Syndrom“ berücksichtigt wurde. Für die so gewonnenen Diagnosegruppen errechneten wir mittels χ^2^-Test nach Pearson Belastungsart und Belastungsstärke und verglichen diese miteinander.

Die statistischen Analysen der erhobenen Daten wurden mit der Software GNU pspp 0.10.2-g654fff und mit IBM SPSS Statistics 25.0 und R 3.6.3 durchgeführt. Für die Analyse aller nominalen Variablen (wie Belastungsart und -stärke, Diagnose, ergriffene Maßnahme oder Geschlecht) wurden Pearson’s χ^2^-Tests verwendet. Bei kleinen Fallzahlen wurden der exakte χ^2^-Test als Monte-Carlo-Variante mit einer 99 %igen Signifikanz und ein exakter Test nach Fisher ^[^*^F]^ eingesetzt. Kritische Signifikanzwerte wurden nach Benjamini und Hochberg (1995) für multiple Vergleiche über alle Analysen hinweg angepasst ^[*BH]^. Die Wirkung kardinalskalierter unabhängiger Variablen (wie Alter und Zahl der Kontakte und Zeitpunkt der Beschwerde) wurden mithilfe einer Probit-Regression erfasst und jeweils mit der GLM (binomial, probit Familie, in R). Die kardinalskalierten abhängigen Variablen „Anzahl der Kontakte“ wurden mithilfe eines T‑Tests erfasst und, da im Levene-Test eine Varianzinhomogenität bestand, mit einer robusten Welch-ANOVA ^[W]^ gerechnet.

Ein positives Votum durch die Ethikkommission der Charité – Universitätsmedizin Berlin (EA1/110/20) lag vor.

## Ergebnisse

Die Stichprobe besteht aus 682 im April 2020 in der Psychiatrischen Institutsambulanz der PUK der Charité im SHK Behandelten, darunter 53,1 % (*n* = 362) Frauen und 46,9 % (*n* = 320) Männer (Tab. 1). Das mittlere Alter betrug 42,70 Jahre (18–87 Jahre; SD = 12,94). 39 % (*n* = 266) der Patient*innen hatten zwei psychische Erkrankungen, wurden also in zwei Diagnosegruppen berücksichtigt. 44,3 % (*n* = 302) hatten spontan Belastungen durch die Pandemie angegeben: 14,5 % (*n* = 99) litten unter der Angst vor dem Virus SARS-CoV‑2, 25,5 % (*n* = 174) unter den Folgen der gesetzlich getroffenen Maßnahmen zur Eindämmung der Pandemie (Kontakt- und Besuchsbeschränkungen, Verlust von Tagesstruktur, finanzielle Einbußen oder Unsicherheit etc.), und 4,3 % (*n* = 29) gaben an, unter beidem zu leiden. 6,3 % (*n* = 43) waren durch pandemieunabhängige Faktoren belastet, 28,2 % (*n* = 192) fühlten sich nicht belastet, und 10,4 % (*n* = 71) fühlten sich in dieser Situation wohler als zuvor (Tab. 2).

**Table 1:** 

	Gesamt	Subjektive Belastung							
		Fehlende Angabe	Nicht belastet	Es geht mir besser	Durch Angst vor Virus	Durch Maßnahmen	Durch beides	Durch etwas anderes	
Gesamt	100 % (*n* = 682)	10,8 % (*n* = 74)	28,2 % *n* = (192)	10,4 % *n* = (71)	14,5 % *n* = (99)	25,5 % *n* = (174)	4,3 % *n* = (29)	6,3 % *n* = (43)	–
Männer	46,9 % (*n* = 320)	11,9 % (*n* = 38)	28,4 % (*n* = 91)	10,6 % (*n* = 34)	12,2 % (*n* = 39)	27,5 % (*n* = 88)	3,4 % (*n* = 11)	5,9 % (*n* = 19)	χ^2^(6) = 4,88 *p* < 0,559 Cramer V = 0,08
Frauen	53,1 % (*n* = 362)	9,9 % (*n* = 36)	27,9 % (*n* = 101)	10,2 % (*n* = 37)	16,6 % (*n* = 60)	23,8 % (*n* = 86)	5,0 % (*n* = 18)	6,6 % (*n* = 24)	
Alter (18–87)	MW 42,7 SD 12,87	42,45 SD = 11,01	43,40 SD 13,40	40,08 SD 12,21	44,72 SD 12,54	41,85 SD 13,07	46,97 SD 17,01	41,67 SD 12,87	F(5, 675) = 1,66 *p* = 0,129
Therapeutische Maßnahme ergriffen	29,01 % (*n* = 181)	3,9 % (*n* = 7)	2,2,% (*n* = 4)	5,5 % (*n* = 10)	23,2 % (*n* = 42)	46,4 % (*n* = 84)	3,3 % (*n* = 6)	15,5,% (*n* = 28)	*χ*^*2*^*(6)* *=* *163,94* *p* *<* *0,0001* *Cramer V* *=* *0,49*
Zahl therapeutischer Kontakte im April 2020	2,03 SD = 1,58 (1–20)	1,46 SD = 0,80 (1–5)	1,67 SD = 0,95 (1–6)	1,97 SD = 1,45 (1–6)	2,02 SD 1,27 (1–6)	2,45 SD = 1,88 (1–16)	1,97 SD = 1,28 (1–8)	3,02 SD = 3,04 (1–20)	*F*^*W*^*(5,152)* *=* *6,501* *P*^*W*^ *<* *0,0001*
Klinische Verschlechterung	60,5 % (*n* = 378)	14,7 % (*n* = 11)	0,5 % (*n* = 1)	47,9 % (*n* = 34)	97,0 % (*n* = 96)	96,0 % (*n* = 167)	96,6 % (*n* = 28)	97,7 % (*n* = 42)	*χ*^*2*^*(12)* *=* *971,23* *p* *<* *0,0001*

Einfaktorielle Varianzanalyse: Die Varianzhomogenität wurde mit Levene getestet. Bei Varianzinhomogenität wurde eine robuste Welch-ANOVA [^W^] gerechnet und anschließend ein Games-Howell-Post-hoc-Test i [^GH^] durchgeführt (Signifikanzniveau 0,05)

Kritisches *p* für multiple Vergleiche nach Benjamini und Hochberg (1995): *p* = 0,03

*χ*^*2*^ χ^2^-Test nach Pearson

**Table 2:** 

Diagnosen nach ICD-10 *n* = 682 Patient*innen *n* = 179 (39 %) davon hatten 2 Diagnosen	Gesamt von 682	Subjektive Belastung							
		Fehlende Angaben	Nicht belastet	Es geht mir besser	Durch Angst vor Virus	Durch Maßnahmen	Durch beides	Durch etwas anderes	Statistische Werte
F1: Abhängigkeit	12,5 % (*n* = 58)	15,3 % (*n* = 13)	29,4 % (*n* = 25)	14,1 % (*n* = 12)	4,7 % (*n* = 4)	29,4 % (*n* = 25)	2,4 % (*n* = 2)	4,7 % (*n* = 4)	χ^2^(6) = 11,140 p*^F^ = 0,053 Cramer V = 0,128
Darunter: Alkoholabhängigkeit, F10	5,3 % (*n* = 36)	13,9 % (*n* = 5)	33,3 % (*n* = 12)	8,3 % (*n* = 3)	5,6 % (*n* = 2)	36,1 % (*n* = 13)	2,8 % (*n* = 1)	0 % (*n* = 0)	χ^2^(6) = 7,119 *p* = 0,310 Cramer V = 0,102
F2: psychotisches Syndrom	23,5 % (*n* = 160)	15,0 % (*n* = 24)	34,4 % (*n* = 55)	13,1 % (*n* = 21)	11,3 % (*n* = 18)	18,4 % (*n* = 29)	0,6 % (*n* = 1)	7,5 % (*n* = 12)	*χ*^*2*^*(6)* *=* *20,498* p*^F^ *=* *0,001* *Cramer V* *=* *0,173*
F3: depressives Syndrom	45,0 % (*n* = 307)	10,4 % (*n* = 32)	28,0 % (*n* = 86)	9,4 % (*n* = 29)	13,7 % (*n* = 42)	27,7 % (*n* = 85)	4,6 % (*n* = 14)	6,2 % (*n* = 19)	χ^2^(6) = 2096 p*^F^ = 0,916 Cramer V = 0,055
F4 – 40/41: ängstliches Syndrom	26,1 % (*n* = 178)	9 % (*n* = 16)	20,8 % (*n* = 37)	7,3 % (*n* = 13)	22,5 % (*n* = 40)	28,7 % (*n* = 51)	7,3 % (*n* = 13)	5,6 % (*n* = 10)	*χ*^*2*^*(6)* *=* *21410* p*^F^ *=* *0,002* *Cramer V* *=* *0,177*
F4 – F42/44/45: sonstige neurotische Syndrome	6,5 % (*n* = 44)	9,1 % (*n* = 4)	20,5 % (*n* = 9)	13,6 % (*n* = 6)	15,9 % (*n* = 7)	22,7 % (*n* = 10)	9,1 % (*n* = 4)	9,1 % (*n* = 4)	χ^2^(6) = 5068 p*^F^ = 0,463 Cramer V = 0,086
PTSD, F43.1	9,7 % (*n* = 66)	6,1 % (*n* = 4)	25,8 % (*n* = 17)	10,6 % (*n* = 7)	12,1 % (*n* = 8)	33,3 % (*n* = 22)	7,6 % (*n* = 5)	4,5 % (*n* = 3)	X^2^(6) = 6,018 *p* = 0,421 Cramer V = 0,138
F6: Persönlichkeitsstörungen	12,0 % (*n* = 82)	8,5 % (*n* = 7)	28,0 % (*n* = 23)	15,9 % (*n* = 13)	3,7 % (*n* = 3)	31,7 % (*n* = 25)	3,7 % (*n* = 3)	8,5 % (*n* = 7)	*χ*^*2*^*(6)* *=* *13,070* p*^F^ *=* *0,018* *Cramer V* *=* *0,138*
Sonstige F0, F7, F8, F9	5,9 % (*n* = 40)	10,0 % (*n* = 4)	27,5 % (*n* = 11)	10,0 % (*n* = 4)	12,5 % (*n* = 5)	27,5 % (*n* = 11)	5,0 % (*n* = 2)	7,5 % (*n* = 3)	χ^2^(6) = 0,417 P*^F^ = 0,996 Cramer V = 0,025

Bei kleiner Subgruppe wurde die Signifikanz [*p*] mit dem exakten Test nach Fisher [p*^F^] berechnet, inklusive Monte-Carlo-Methode (Konfidenzniveau 99 %). Kritisches *p* für multiple Vergleiche nach Benjamini und Hochberg (1995): *p* = 0,0055

*χ*^*2*^ χ^2^-Test nach Pearson

Bei 60,5 % (*n* = 378) war von den Behandelnden eine klinische Verschlechterung der Symptomatik beschrieben worden, bei 20,8 % (*n* = 142) eine schwere, die pathologische Verhaltensänderungen beinhaltete (Tab. 3). 29,1 % (*n* = 181) aller Patient*innen hielten wir für so schwer belastet, dass wir gegensteuernde Maßnahmen ergriffen: Von diesen waren 72,9 % (*n* = 132) von der Pandemie belastet, 15,5 % (*n* = 28) durch anderweitige Faktoren und 7,7 % (*n* = 14) derer, die sich subjektiv nicht belastet fanden. Die klinisch leicht oder schwer Belasteten erhielten im April signifikant mehr therapeutische Kontakte als die nicht Belasteten (adjustierter kritischer *p*-Wert 0,03 nach Benjamini und Hochberg; MW = 2,03 Kontakte; SD = 1,58, Welch-Test F[5, 152.196] = 6,501, *p*^W^ < 0,0001), der Games-Howell-Post-hoc-Test zeigte hier einen signifikanten Unterschied für die unter den Maßnahmen Leidenden im Vergleich zu den nicht Leidenden (0,782, *p* < 0,0001, 95 %-CI [0,33, 1,24]; Tab. 1). 18 Behandelte mussten aufgrund der Schwere der Symptomatik in stationäre Behandlung weitergeleitet werden: Von diesen waren 8 durch die Schutzmaßnahmen und ihre Folgen und 6 durch pandemieunabhängige Faktoren betroffen.

**Table 3:** 

Diagnosen nach ICD10 *n* = 682 Patient*innen *n* = 179 (39 %) davon hatten 2 Diagnosen	Klinische Verschlechterung				
	Gesamt	Nicht	Leicht	Schwer	Statistische Werte
F1: Abhängigkeit	100 % (*n* = 78)	44,9 % (*n* = 35)	34,9 % (*n* = 27)	20,5 % (*n* = 16)	χ^2^(2) = 1111 p*^F^ = 0,372
Darunter: Alkoholabhängigkeit, F10	100 % (*n* = 33)	42,2 % (*n* = 14)	27,3 % (*n* = 9)	30,3 % (*n* = 10)	χ^2^(2) = 1981 *p* = 0,576
F2: psychotisches Syndrom	100 % (*n* = 140)	56,0 % (*n* = 77)	27,1 % (*n* = 38)	17,9 % (*n* = 25)	*χ*^*2*^*(2)* *=* *18.454* p*^F^ *<* *0,001*
F3: depressives Syndrom	100 % (*n* = 263)	36,0 % (*n* = 102)	39,9 % (*n* = 113)	24,0 % (*n* = 48)	χ^2^(2) = 2478 p*^F^ = 0,290
F4 – 40/41: ängstliches Syndrom	100 % (*n* = 164)	28,0 % (*n* = 46)	43,3 % (*n* = 71)	28,7 % (*n* = 47)	*χ*^*2*^*(2)* *=* *12465* p*^F^ *=* *0,002*
F4 – 44/45: sonstige neurotische Syndrome	100 % (*n* = 31)	36,6 % (*n* = 15)	34,1 % (*n* = 14)	29,3 % (*n* = 12)	χ^2^(2) = 1064 p*^F^ = 0,587
PTSD, F43.1	100 % (*n* = 61)	32,8 % (*n* = 20)	41,0 % (*n* = 25)	26,2 % (*n* = 16)	χ^2^(2) = 1,290 *p* = 0,524
F6: Persönlichkeitsstörungen	100 % (*n* = 78)	46,2 % (*n* = 36)	35,9 % (*n* = 28)	17,9 % (*n* = 14)	χ^2^(2) = 2017 p*^F^ = 0,365
Sonstige F0, F7, F8, F9	100 % (*n* = 35)	36,1 % (*n* = 13)	44,4 % (*n* = 16)	19,4 % (*n* = 7)	χ^2^(2) = 0,734 P*^F^ = 0,593
Frauen	100 % (*n* = 334)	38,3 % (*n* = 128)	40,1 % (*n* = 134)	21,6 % (*n* = 72)	χ^2^(2) = 1,68 P*^F^ = 0,432
Männer	100 % (*n* = 290)	40,7 % (*n* = 118)	35,2 % (*n* = 102)	24,1 % (*n* = 79)	
Gesamt	100 % (*n* = 624)	39,4 % (*n* = 246)	37,8 % (*n* = 236)	22,7 % (*n* = 142)	Keine Angaben: *n* = 58

Bei kleiner Subgruppe wurde die Signifikanz [*p*] mit dem exakten Test nach Fisher [p*^F^] berechnet, inclusive Monte Carlo-Methode (Konfidenzniveau 99 %). Kritisches *p* für multiple Vergleiche nach Benjamini und Hochberg (1995): *p* = 0,0055

39 % der Patient*innen wiesen Symptome auf, die in zwei unterschiedlichen Syndromgruppen erfasst wurden

*χ*^*2*^ χ^2^-Test nach Pearson

Für Schwere und Art der Belastung bestanden signifikante Zusammenhänge mit den vorbestehenden Diagnosen (χ^2^[154] = 252,938, p^*F^ = 0,003, Cramer’s V = 0,23) (Tab. 2). Für die folgenden Post-hoc-Vergleiche ist der kritische *p*-Werte nach Benjamini und Hochberg adjustiert (*p* = 0,0055). Insgesamt wurde deutlich, dass Angsterkrankte signifikant häufiger Angst vor einer Virusinfektion hatten als vor anderen Belastungen (χ^2^[6] = 21,410, p*^F^ = 0,002, Cramer V = 0,177). Bei ihnen wurden auch signifikant mehr klinische Verschlechterungen beobachtet (χ^2^[1] = 12,02, *p* < 0,001). In den Aufzeichnungen wurde deutlich, dass diese sich häufig zu einer für eine Ansteckung besonders gefährdeten Risikogruppe rechneten, ohne aus medizinischer Sicht zu einer zu gehören. Sie behielten die Isolationsmaßnahmen häufig weit über das gesetzlich angeordnete Maß hinaus bei.

Psychotisch Erkrankte gaben signifikant weniger Belastung an und häufiger das Gefühl, es gehe besser als zuvor (χ^2^[6] = 20,498, p*^F^ = 0,001, Cramers V = 0,137) und es bestand auch signifikant seltener eine beobachtete Verschlechterung der Psychopathologie (χ^2^[1] = 18,339, *p* < 0,0001). Personen mit Persönlichkeitsstörungen litten signifikant häufiger unter Lockdown- und Hygienemaßnahmen und signifikant seltener an Angst vor Infektionen als anders Erkrankte (χ^2^[6] = 13,070, p*^F^ = 0,018, Cramers V = 0,138).

Für diejenigen mit anderen Diagnosen, wie z. B. depressiv, sucht- oder posttraumatisch Erkrankte, konnten wir im Vergleich zu den anderen Diagnosegruppen keine signifikanten Unterschiede in den Belastungen feststellen.

Als besonders prägnante Beschwerden wurden völlige Selbstisolation, körperliche Symptomatik, Impulskäufe, Retraumatisierung, Trinkrezidive, Familienstreitigkeiten und einmal ein Verschwörungswahn beobachtet. 10,8 % (*n* = 74) der Behandelten hatten sich nicht spontan zu der subjektiven Art ihrer Belastung geäußert – oder dies war nicht dokumentiert worden, und in 8,20 % (*n* = 56) der Fälle war aufgrund der Dokumentation nicht zu entscheiden, ob sich die klinische Symptomatik im Vergleich zu den Vorkontakten verändert hatte.

Für Geschlecht bestanden weder bezüglich Stärke (χ^2^[2] = 0,95, p*^F^ = 0,622) noch bezüglich Art der Belastung (χ^2^[6] = 4,88, p*^F^ = 0,559) signifikante Unterschiede. Das gleiche gilt für die Variable „Alter“ bezüglich Stärke (Z = 0,066, *p* = 0,947) und Art der Belastung (Z = −0,553, *p* = 0,580), und auch für die Variable „Zeitpunkt der Äußerung“ – vor oder nach begonnener Lockerung der Maßnahmen – bezüglich der Stärke (Z = −0,200, *p* = 0,841) und der Art der Belastung (Z = 0,649, *p* = 0,516; Tab. 1). Über die Art der klinischen Dekompensation konnten wir bei nur 4,96 % (*n* = 34) eine konkrete Beschreibung sichern, darunter 5 Trinkrezidive, 5 eskalierende Familienkonflikte und 2 Retraumatisierungen.

Neun Patient*innen wurden aufgrund der aktuellen psychischen Belastung erstmals in psychiatrisch-psychotherapeutische Behandlung aufgenommen; von diesen litten 6 unter eine depressiven, 2 unter einer ängstlichen Symptomatik und eine Person an einer Alkoholerkrankung.

## Diskussion

Die Ergebnisse unserer Studie bestätigen diagnoseabhängige signifikante Unterschiede in der Zunahme krankheitswertiger Symptomatik bei psychisch erkrankten Menschen in den ersten Wochen der COVID-19-Pandemie und der von ihnen subjektiv wahrgenommenen Ursache der Belastung. Bei 60,5 % unserer Stichprobe beobachteten wir eine klinische Verschlechterung im Vergleich zum unmittelbaren Vorkontakt; 44,3 % (*n* = 302) gaben von sich aus spontan an, dass sie diese Verschlechterung subjektiv als durch die Pandemie bedingt erlebten. Anders als in einer großen Stichprobe chinesischer Allgemeinbevölkerung beobachtet, konnten wir in unserer Stichprobe für Frauen keine signifikant häufigere Angst vor dem Virus beschreiben [39]. Jedoch konnten wir die Beobachtung, dass die subjektive Wahrnehmung eigener schlechter Gesundheit den psychischen Stress erhöht [5], bei vielen Menschen mit einer Angsterkrankung bestätigen.

Während in Italien für die psychisch gesunde ältere Allgemeinbevölkerung eine größere Resilienz gegenüber einer psychischen Belastung durch die Pandemie beschrieben wurde [7] und in Frankreich eher eine höhere Vulnerabilität für ältere Personen mit psychischer Vorerkrankung identifiziert wurde [6], sahen wir in unserer Stichprobe keinen signifikanten Zusammenhang zwischen Lebensalter und Art oder Stärke der Belastung durch die Pandemie. Offenbar bestehen in Deutschland im Vergleich zu anderen Ländern sowohl bezüglich der sozialen Sicherheit schwer psychisch Erkrankter als auch körperlicher Gesundheit keine so gravierenden altersabhängigen Unterschiede in der subjektiv erlebten Gefährdung durch Vorerkrankungen.

Auch konnten wir keine geschlechtsabhängigen Unterschiede in Zusammenhang mit Art oder Stärke der Belastung bestätigen [7, 8]. Möglicherweise hängt das damit zusammen, dass in unserer Stichprobe – mit einem hohen Anteil chronisch psychisch erkrankter Menschen – rollenspezifische Unterschiede eine geringere Rolle spielen als in der Allgemeinbevölkerung (z. B. bezüglich Versorgung von Kindern, Sorge um Arbeitsplatz etc.).

Eine signifikante Verschlechterung bei der Gruppe derjenigen, die unter einer PTSD litten, konnten wir nicht beobachten: Lediglich 2 Personen mit vorbestehender PTSD erlitten durch Pandemiemaßnahmen eine Retraumatisierung. Von den 9 uns neu zugewiesenen Patient*innen litt keine*r unter einer PTSD. Die im Vergleich zu den Studien aus Italien, China, USA und Tunesien [7, 11, 20, 40] geringe Bedeutung einer Traumatisierung in unserer Studienpopulation psychisch erkrankter Menschen in Deutschland könnte auch im Zusammenhang damit stehen, dass in weiten Teilen Deutschlands bislang ein blander Pandemieverlauf erlebt wurde.

Interessant bleibt die Frage, was die psychische Widerstandsfähigkeit der Menschen – hier also insbesondere psychisch erkrankte Menschen – befähigt, schwierige Lebenssituationen ohne anhaltende Beeinträchtigung zu überstehen. So empfehlen mehrere Autoren, die Resilienzfähigkeit dieser Subpopulation zu testen [24] und zu fördern [38]. Für das gute Überstehen einer schweren psychischen Belastung wurden für die gesunde Allgemeinbevölkerung verschiedene angeborene und erworbene Faktoren, u. a. aktiv bleiben, Belastungen als Wachstumschance begreifen können, Optimismus, Sinn für Humor, Arbeit und Liebe genannt [19]. Ein gutes Selbstbild helfe, übrigens ebenso wie ein gemeinsamer Widerstand gegen einen äußeren Feind, eine Quarantäne zu überstehen [5]: Alles Eigenschaften, die psychisch Erkrankten zumindest in einer Krankheitsphase nicht oder vermindert zur Verfügung stehen.

Aus Italien wurde berichtet, dass diejenigen, die sich in Quarantäne befunden hatten, signifikant weniger Stresssymptomatik hatten als die restliche Allgemeinbevölkerung – allerdings hatten diese während der Quarantäne auch psychologische Unterstützung erhalten [23]. Stress sei mit sozialer Unterstützung sehr viel besser zu verarbeiten als ohne, dyadisches Coping in der (intakten) Partnerbeziehung am hilfreichsten. Unter den auf Intensivstationen in den USA Beschäftigte wurden feste Paare gegenseitiger Unterstützung geschaffen, ein „peer support“ ähnlich einem in der US-Armee üblichen, den „battle buddies“ [1]. Die Krisentelefone [36] oder anonymen telefonischen Gesprächsangebote für Senior*innen [29] sind vorhandene Hilfsangebote, die psychologische Unterstützung anbieten und Vereinsamten telefonische Gesprächspartner*innen vermitteln können. Für chronisch psychiatrisch Erkrankte, die oft sehr viel eingeschränkter sind in der Fähigkeit, Hilfe aufzusuchen, Vertrauen zu bilden, Kontakt aufzunehmen, und die im Alltag sehr oft allein leben, sind diese Angebote nur selten realisierbar.

Hier wurde die Bedeutung der therapeutischen Beziehung deutlich [30], denn wir konnten viele Betroffene durch die Erhöhung von Frequenz und Intensität der therapeutischen Gesprächsangebote stabilisieren. Allerdings forderte dieses Vorgehen sehr viel Energie und Zeit. Deutlich wird, dass der ambulante psychiatrische Sektor in Pandemiezeiten ebenfalls als dringend personell zu verstärkender Gesundheitsbereich gedacht werden sollte. Zur Förderung der psychischen Stabilität der Betroffenen könnte das Prinzip des partnerschaftlichen „peer support“ eine Methode oder Maßnahme sein, die in der psychiatrischen Versorgung chronisch Erkrankter künftig mitgedacht werden sollte.

Neben dem unterstützenden sozialen Kontakt werden noch einige weitere Faktoren genannt, die psychische Widerstandsfähigkeit in Pandemiezeiten förderten: geringes Risiko sich anzustecken, große Hoffnung auf das eigene Überleben, hohes Vertrauen in Ärzte und Wissen über Virus und Erkrankung [34, 40]. In vertrauensvollem Setting vermittelte valide Kenntnisse über Ansteckungswege und sinnvolle Schutzmaßnahmen, Krankheitsverlauf bei Ansteckung [42] sowie Anleitung gesundheitsförderliche Maßnahmen („unbedingt einmal am Tag 0,5 h Spazieren gehen“) helfe, weniger durch das Geschehen belastet zu werden.

Interessant bleibt, dass es in unserer Stichprobe einige Menschen gab, die die soziale Distanzierung subjektiv als angenehm empfanden und es genossen, mehr Ruhe zu haben, weniger Lärm und Unruhe auf den Straßen, keine belastenden Anfragen von Jobcenter und anderen Ämtern – signifikant häufiger waren dies Menschen, die eine Psychose erlebt hatten oder erleben. Bei ihnen war auch von den Behandelnden im Aktenverlauf eine geringere klinische Belastung dokumentiert worden. Anderseits waren auch 8 von den 18 Patient*innen, die im April 2020 so schwer dekompensierten, dass sie stationär aufgenommen werden mussten, psychotisch vorerkrankt. Hier sind Vergleichsbeobachtungen zu einem Äquivalenzzeitraum ohne Pandemie notwendig, um dies abschließend beurteilen zu können. Dasselbe gilt für die von uns in diesem Zeitrahmen beobachteten Trinkrezidive bei 5 von 36 zuvor abstinenten alkoholabhängig Erkrankten.

Unsere Untersuchungsergebnisse sind dadurch limitiert, dass wir in 10,8 % (*n* = 74) der Fälle die Art und in 8,20 % (*n* = 58) der Fälle die Schwere der Belastung nicht ermitteln konnten. Über die Art der klinischen Dekompensation konnten wir aufgrund fehlender Vergleichsdaten keine statistisch valide Aussage machen, hier sind weitere Untersuchungen erforderlich. Eine weitere Limitation unserer Studie ist, dass sie nur einen sehr kurzen Zeitraum berücksichtigt. Da die Untersuchung retrospektiv erfolgte, konnten wir auch nur die deutlich schriftlich dokumentierten Beschwerden der Betroffenen aufnehmen. Auch konnten wir keine standardisierten Fragebogeninstrumente verwenden, weshalb sich unsere Daten nicht mit den bisherigen Studien – z. B. in der Allgemeinbevölkerung – vergleichen lassen. Ebenso konnten in unserer Datenbank einige weitere, potenziell relevante Faktoren – wie z. B. sozioökonomischer Status und kultureller Hintergrund – nicht quantifiziert werden, Aspekte, die in künftigen Studien berücksichtigt werden könnten. Auch sind weitere Studien mit größeren Stichproben zur Beurteilung des längeren Verlaufes sowohl bei psychisch Erkrankten als auch der pandemiebedingten Entwicklung psychischer Erkrankungen in der bislang gesunden Bevölkerung erforderlich.

Durch unsere Studie wird deutlich, dass das weitere Erforschen und Entwickeln von Maßnahmen zur Förderung von der Resilienz bei psychisch schwer Erkrankten vordringlich bleibt. Themen wie „peer support“ könnten weitergedacht, evaluiert und ggf. implementiert werden. Zudem gibt unsere Studie wertvolle Hinweise darauf, welche Patient*innengruppen besonders bei künftigen Pandemien durch präventive Maßnahmen besser geschützt werden können.

## Schlussfolgerungen für die Praxis

- Psychiatrisch Erkrankte sind bei erneuten seuchengesetzlichen Kontaktbeschränkungen besonders zu berücksichtigen, z. B. indem soziale therapeutische Angebote auch unter rigiderem Hygienekonzept zeitnah möglich gemacht werden (Spaziergänge, Gruppen im Freien, sportliche Aktivitäten ect.).
- Bei erneutem Infektionsgeschehen und Lockdown sollte der Kontakt zu psychisch Vorerkrankten aktiv gesucht werden (telefonisch, per Video gestützt, analog, ggf. Hausbesuche).
- Eine personelle Verstärkung ambulanter psychiatrischer Einrichtungen sollte in der medizinischen Planung bei globalen Bedrohungen berücksichtigt werden, denn in der sog. ersten Welle der COVID-19-Pandemie wurde mit 60,5 % ein großer Teil der Patient*innen einer psychiatrischen Ambulanz klinisch belastet, bei 29,1 % der Behandelten wurden therapeutische Maßnahmen erforderlich.
- Resilienzforschung und -förderung für psychisch Erkrankte ist dringend erforderlich.
